# Supplementary material for: Genomic evidence for the degradation of terrestrial organic matter by pelagic Arctic Ocean Chloroflexi bacteria
Source: Commun Biol. 2018 Jul 5;1:90. doi: 10.1038/s42003-018-0086-7 (PMC6123686; doi:10.1038/s42003-018-0086-7)
Supplement: Supplementary file 1 — Supplementary Information [file 42003_2018_86_MOESM1_ESM.pdf]

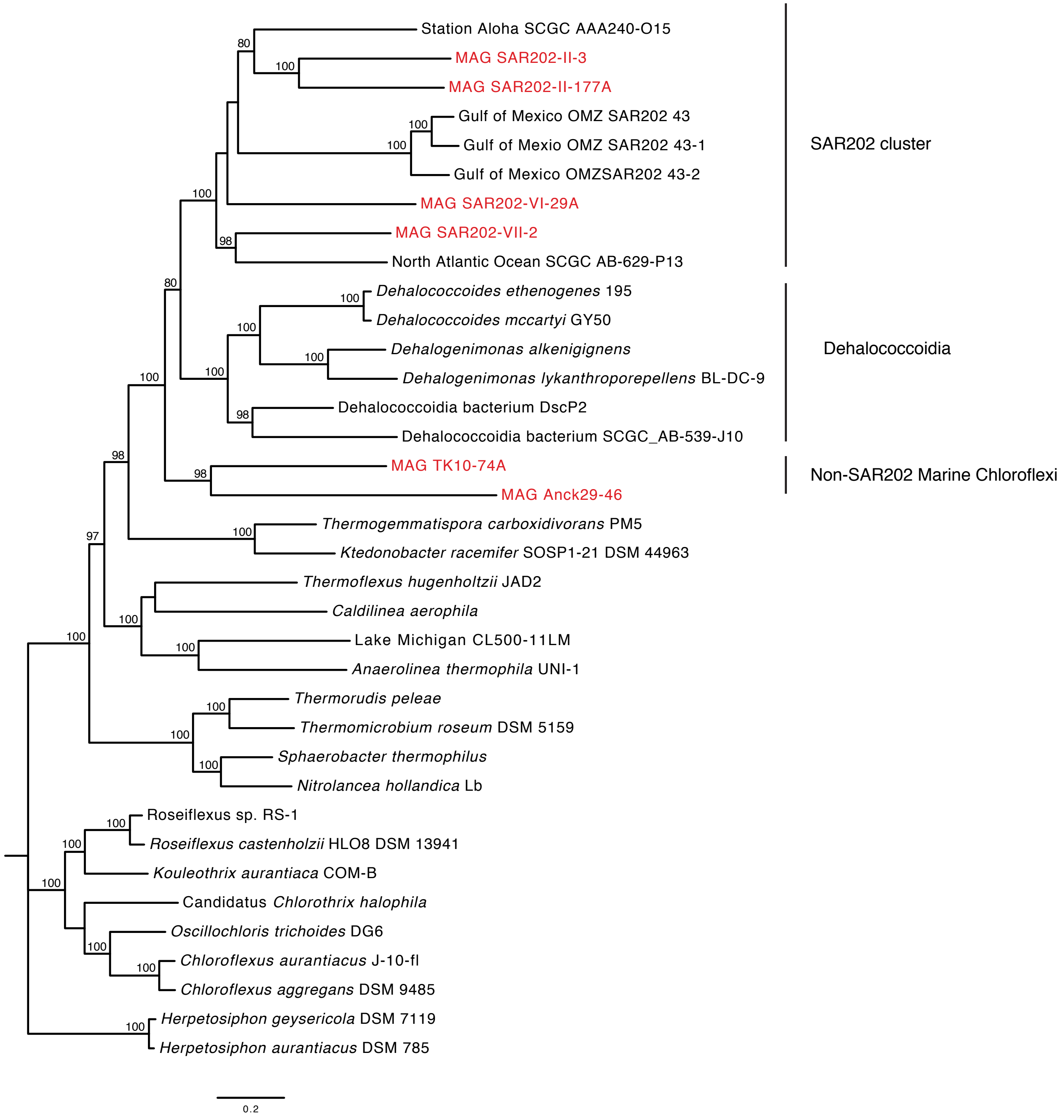

Station Aloha SCGC AAA240-O15
MAG SAR202-II-3
MAG SAR202-II-177A
Gulf of Mexico OMZ SAR202 43
Gulf of Mexio OMZ SAR202 43-1
Gulf of Mexico OMZSAR202 43-2
MAG SAR202-VI-29A
MAG SAR202-VII-2
North Atlantic Ocean SCGC AB-629-P13
SAR202 cluster
Dehalococcoides ethenogenes 195
Dehalococcoides mccartyi GY50
Dehalogenimonas alkenigignens
Dehalogenimonas lykanthroporepellens BL-DC-9
Dehalococcoidia bacterium DscP2
Dehalococcoidia bacterium SCGC_AB-539-J10
Dehalococcoidia
MAG TK10-74A
MAG Anck29-46
Non-SAR202 Marine Chloroflexi
Thermogemmatispora carboxidivorans PM5
Ktedonobacter racemifer SOSP1-21 DSM 44963
Thermoflexus hugenholtzii JAD2
Caldilinea aerophila
Lake Michigan CL500-11LM
Anaerolinea thermophila UNI-1
Thermorudis peleae
Thermomicrobium roseum DSM 5159
Sphaerobacter thermophilus
Nitrolancea hollandica Lb
Roseiflexus sp. RS-1
Roseiflexus castenholzii HLO8 DSM 13941
Kouleothrix aurantiaca COM-B
Candidatus Chlorothrix halophila
Oscillochloris trichoides DG6
Chloroflexus aurantiacus J-10-fl
Chloroflexus aggregans DSM 9485
Herpetosiphon geysericola DSM 7119
Herpetosiphon aurantiacus DSM 785
0.2

Supplementary figure 1. A concatenated protein phylogeny of Chloroflexi genomes including 30 Chloroflexi reference genomes and the 6 Canada Basin Chloroflexi MAGs. This is a maximum likelihood tree based on concatenated sequence alignment of fifty amino acid sequences (representing of 14,815 amino acid positions), present in at least 34 of the 36 Chloroflexi genomes. Bootstrap values are based on 100 replicates.

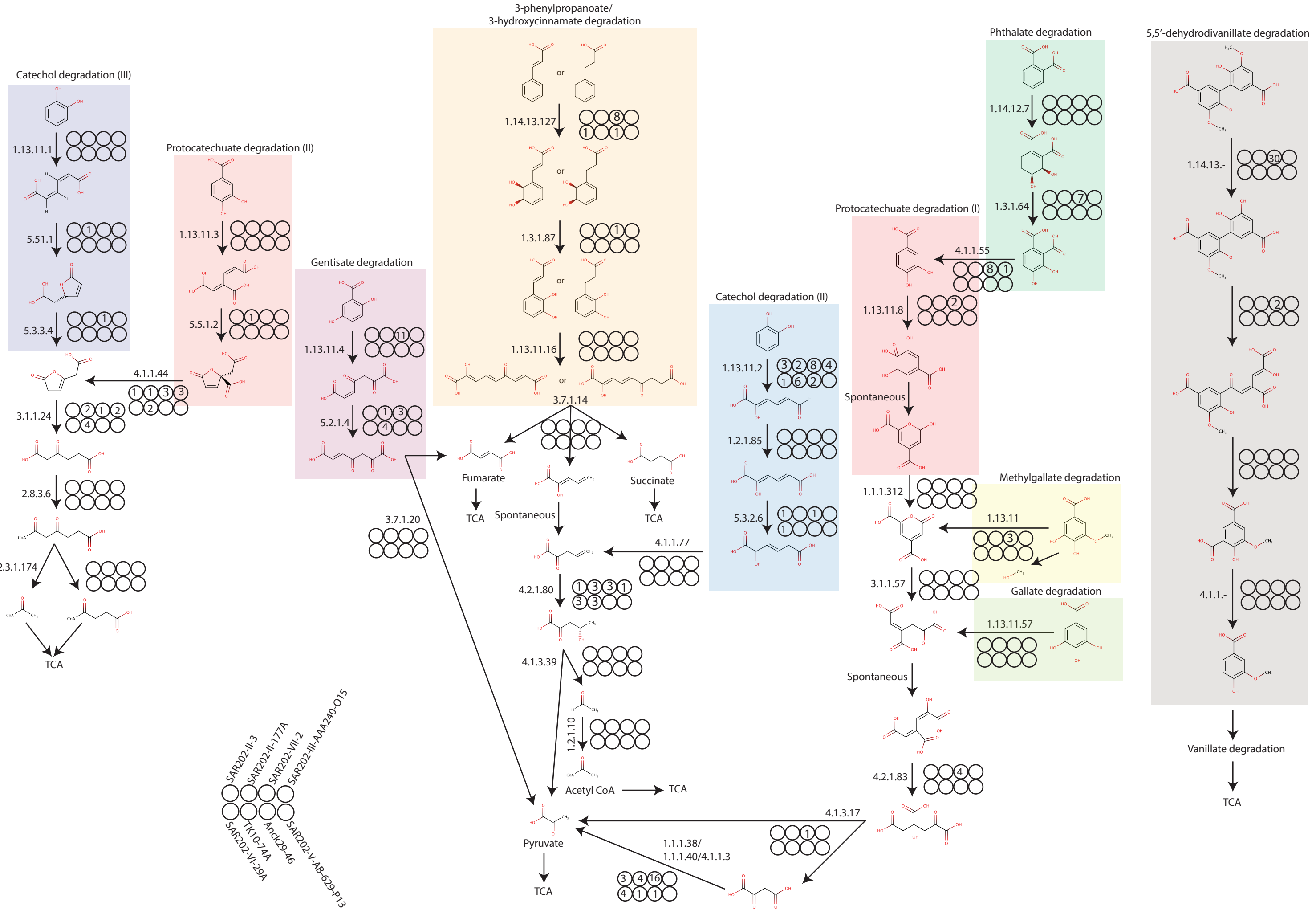

3-phenylpropanoate/
3-hydroxycinnamate degradation
Catechol degradation (III)
Protocatechuate degradation (II)
Gentisate degradation
Catechol degradation (II)
Protocatechuate degradation (I)
Phthalate degradation
5,5′-dehydrodivanillate degradation
Methylgallate degradation
Gallate degradation
1.13.11.1
5.51.1
5.3.3.4
1.13.11.3
5.5.1.2
4.1.1.44
3.1.1.24
2.8.3.6
2.3.1.174
TCA
1.13.11.4
5.2.1.4
1.14.13.127
1.3.1.87
1.13.11.16
or
3.7.1.14
Fumarate
Succinate
Spontaneous
3.7.1.20
4.1.1.77
4.2.1.80
4.1.3.39
1.2.1.10
Acetyl CoA
Pyruvate
4.1.3.17
1.1.1.38/
1.1.1.40/4.1.1.3
1.13.11.2
1.2.1.85
5.3.2.6
1.14.12.7
1.3.1.64
4.1.1.55
1.13.11.8
1.1.1.312
1.13.11
3.1.1.57
1.13.11.57
4.2.1.83
1.14.13.-
4.1.1.-
Vanillate degradation
SAR202-II-3
SAR202-II-177A
SAR202-VII-2
SAR202-III-AAA240-O15
SAR202-VI-29A
TK10-74A
Anck29-46
SAR202-V-AB-629-P13

Supplementary figure 2. Aromatic compound degradation pathways identified in the Arctic Ocean and deep ocean Chloroflexi genomes. Circles represent the six Arctic Ocean MAGs and two deep ocean SAGs. The values in the circles represent the number of homologs identified in each respective genome. These reference metabolic pathways from MetaCyc were populated with proteins based on functional annotations available at the Integrated Microbial Genomes database.

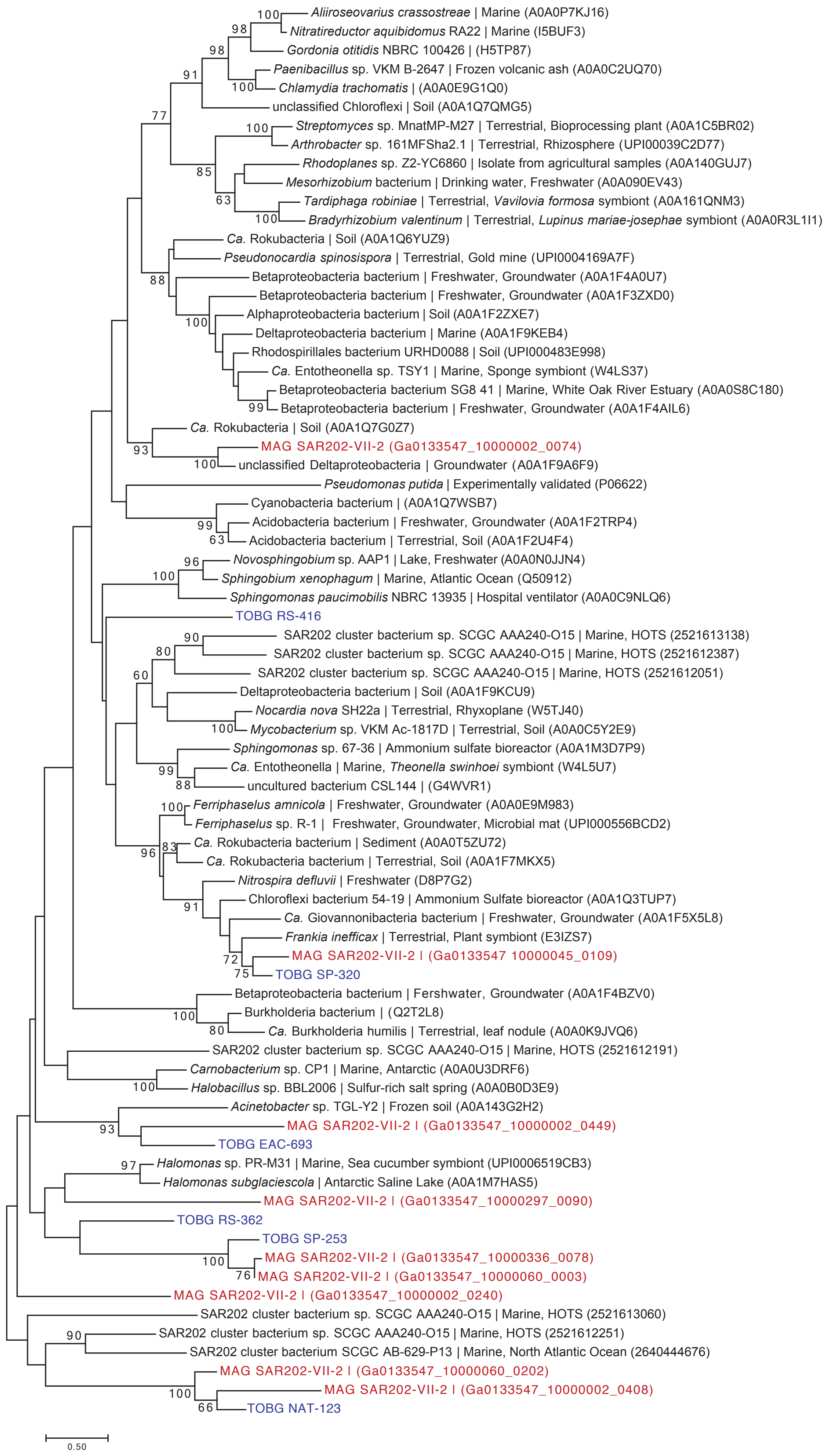

Aliiroseovarius crassostreae | Marine (A0A0P7KJ16)
Nitratireductor aquibidomus RA22 | Marine (I5BUF3)
Gordonia otitidis NBRC 100426 | (H5TP87)
Paenibacillus sp. VKM B-2647 | Frozen volcanic ash (A0A0C2UQ70)
Chlamydia trachomatis | (A0A0E9G1Q0)
unclassified Chloroflexi | Soil (A0A1Q7QMG5)
Streptomyces sp. MnatMP-M27 | Terrestrial, Bioprocessing plant (A0A1C5BR02)
Arthrobacter sp. 161MFSha2.1 | Terrestrial, Rhizosphere (UPI00039C2D77)
Rhodoplanes sp. Z2-YC6860 | Isolate from agricultural samples (A0A140GUJ7)
Mesorhizobium bacterium | Drinking water, Freshwater (A0A090EV43)
Tardiphaga robiniae | Terrestrial, Vavilovia formosa symbiont (A0A161QNM3)
Bradyrhizobium valentinum | Terrestrial, Lupinus mariae-josephae symbiont (A0A0R3L1I1)
Ca. Rokubacteria | Soil (A0A1Q6YUZ9)
Pseudonocardia spinosispora | Terrestrial, Gold mine (UPI0004169A7F)
Betaproteobacteria bacterium | Freshwater, Groundwater (A0A1F4A0U7)
Betaproteobacteria bacterium | Freshwater, Groundwater (A0A1F3ZXD0)
Alphaproteobacteria bacterium | Soil (A0A1F2ZXE7)
Deltaproteobacteria bacterium | Marine (A0A1F9KEB4)
Rhodospirillales bacterium URHD0088 | Soil (UPI000483E998)
Ca. Entotheonella sp. TSY1 | Marine, Sponge symbiont (W4LS37)
Betaproteobacteria bacterium SG8 41 | Marine, White Oak River Estuary (A0A0S8C180)
Betaproteobacteria bacterium | Freshwater, Groundwater (A0A1F4AIL6)
Ca. Rokubacteria | Soil (A0A1Q7G0Z7)
MAG SAR202-VII-2 (Ga0133547_10000002_0074)
unclassified Deltaproteobacteria | Groundwater (A0A1F9A6F9)
Pseudomonas putida | Experimentally validated (P06622)
Cyanobacteria bacterium | (A0A1Q7WSB7)
Acidobacteria bacterium | Freshwater, Groundwater (A0A1F2TRP4)
Acidobacteria bacterium | Terrestrial, Soil (A0A1F2U4F4)
Novosphingobium sp. AAP1 | Lake, Freshwater (A0A0N0JJN4)
Sphingobium xenophagum | Marine, Atlantic Ocean (Q50912)
Sphingomonas paucimobilis NBRC 13935 | Hospital ventilator (A0A0C9NLQ6)
TOBG RS-416
SAR202 cluster bacterium sp. SCGC AAA240-O15 | Marine, HOTS (2521613138)
SAR202 cluster bacterium sp. SCGC AAA240-O15 | Marine, HOTS (2521612387)
SAR202 cluster bacterium sp. SCGC AAA240-O15 | Marine, HOTS (2521612051)
Deltaproteobacteria bacterium | Soil (A0A1F9KCU9)
Nocardia nova SH22a | Terrestrial, Rhyxoplane (W5TJ40)
Mycobacterium sp. VKM Ac-1817D | Terrestrial, Soil (A0A0C5Y2E9)
Sphingomonas sp. 67-36 | Ammonium sulfate bioreactor (A0A1M3D7P9)
Ca. Entotheonella | Marine, Theonella swinhoei symbiont (W4L5U7)
uncultured bacterium CSL144 | (G4WVR1)
Ferriphaselus amnicola | Freshwater, Groundwater (A0A0E9M983)
Ferriphaselus sp. R-1 | Freshwater, Groundwater, Microbial mat (UPI000556BCD2)
Ca. Rokubacteria bacterium | Sediment (A0A0T5ZU72)
Ca. Rokubacteria bacterium | Terrestrial, Soil (A0A1F7MKX5)
Nitrospira defluvii | Freshwater (D8P7G2)
Chloroflexi bacterium 54-19 | Ammonium Sulfate bioreactor (A0A1Q3TUP7)
Ca. Giovannonibacteria bacterium | Freshwater, Groundwater (A0A1F5X5L8)
Frankia inefficax | Terrestrial, Plant symbiont (E3IZS7)
MAG SAR202-VII-2 l (Ga0133547 10000045_0109)
TOBG SP-320
Betaproteobacteria bacterium | Fershwater, Groundwater (A0A1F4BZV0)
Burkholderia bacterium | (Q2T2L8)
Ca. Burkholderia humilis | Terrestrial, leaf nodule (A0A0K9JVQ6)
SAR202 cluster bacterium sp. SCGC AAA240-O15 | Marine, HOTS (2521612191)
Carnobacterium sp. CP1 | Marine, Antarctic (A0A0U3DRF6)
Halobacillus sp. BBL2006 | Sulfur-rich salt spring (A0A0B0D3E9)
Acinetobacter sp. TGL-Y2 | Frozen soil (A0A143G2H2)
MAG SAR202-VII-2 l (Ga0133547_10000002_0449)
TOBG EAC-693
Halomonas sp. PR-M31 | Marine, Sea cucumber symbiont (UPI0006519CB3)
Halomonas subglaciescola | Antarctic Saline Lake (A0A1M7HAS5)
MAG SAR202-VII-2 l (Ga0133547_10000297_0090)
TOBG RS-362
TOBG SP-253
MAG SAR202-VII-2 l (Ga0133547_10000336_0078)
MAG SAR202-VII-2 l (Ga0133547_10000060_0003)
MAG SAR202-VII-2 l (Ga0133547_10000002_0240)
SAR202 cluster bacterium sp. SCGC AAA240-O15 | Marine, HOTS (2521613060)
SAR202 cluster bacterium sp. SCGC AAA240-O15 | Marine, HOTS (2521612251)
SAR202 cluster bacterium SCGC AB-629-P13 | Marine, North Atlantic Ocean (2640444676)
MAG SAR202-VII-2 l (Ga0133547_10000060_0202)
MAG SAR202-VII-2 l (Ga0133547_10000002_0408)
TOBG NAT-123
0.50

Supplementary figure 3. Phylogenetic analysis of predicted catechol 2,3-dioxygenase (pfam 00903) protein sequences identified in SAR202-VII-2 . This is a maximum likelihood tree based the alignment of catechol 2,3-dioxygenase sequences identified in SAR202-VII-2, UniRef90 sequences and Chloroflexi MAGs constructed from the TARA Oceans dataset. Bootstrap values of >60% are shown (100 replicates). SAR202-VII-2 sequences are shown in red while sequences originating from the TARA Oceans MAGs are shown in blue.

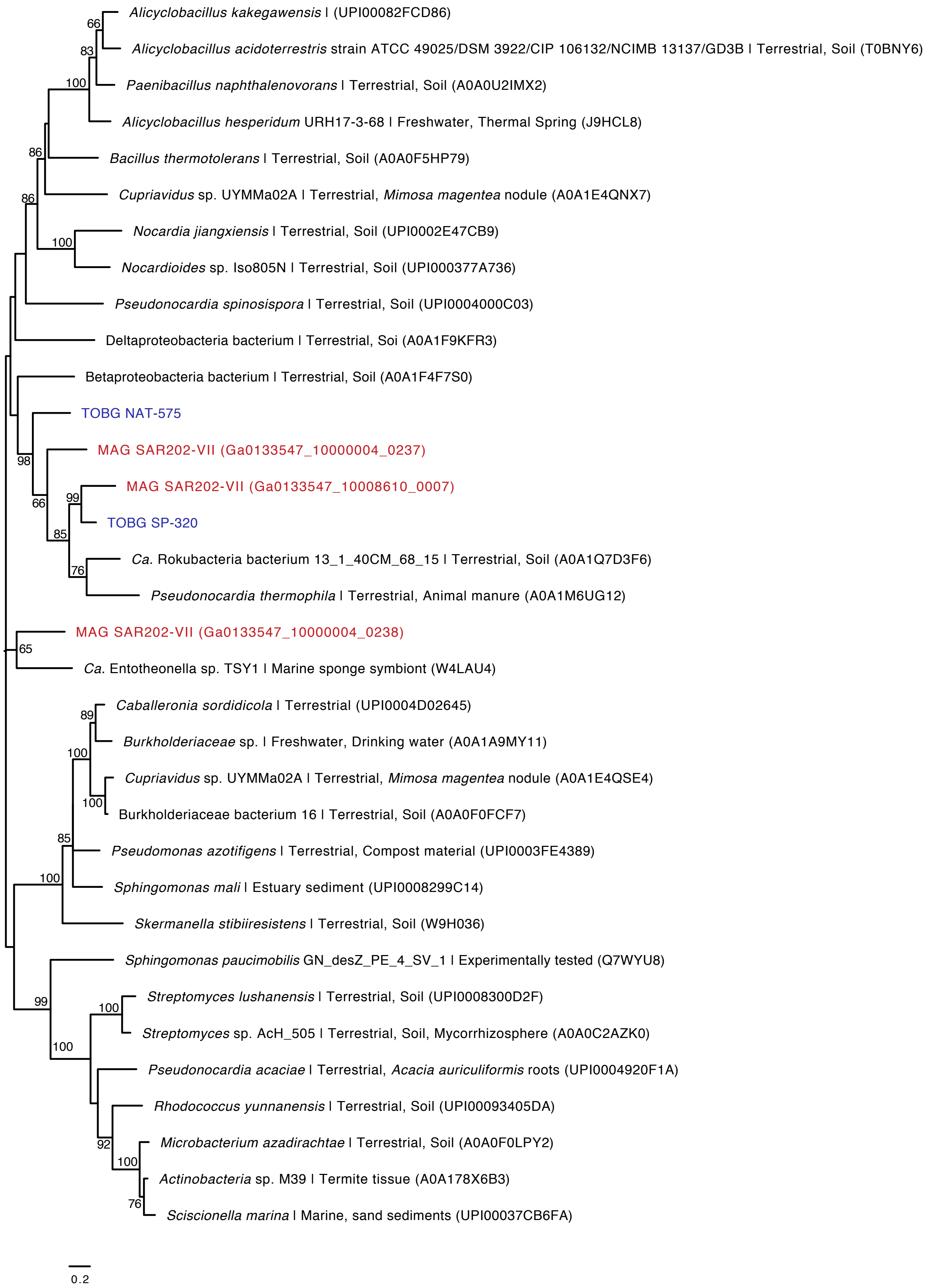
Alicyclobacillus kakegawensis | (UPI00082FCD86)
Alicyclobacillus acidoterrestris strain ATCC 49025/DSM 3922/CIP 106132/NCIMB 13137/GD3B | Terrestrial, Soil (T0BNY6)
Paenibacillus naphthalenovorans | Terrestrial, Soil (A0A0U2IMX2)
Alicyclobacillus hesperidum URH17-3-68 | Freshwater, Thermal Spring (J9HCL8)
Bacillus thermotolerans | Terrestrial, Soil (A0A0F5HP79)
Cupriavidus sp. UYMMa02A | Terrestrial, Mimosa magentea nodule (A0A1E4QNX7)
Nocardia jiangxiensis | Terrestrial, Soil (UPI0002E47CB9)
Nocardioides sp. Iso805N | Terrestrial, Soil (UPI000377A736)
Pseudonocardia spinosispora | Terrestrial, Soil (UPI0004000C03)
Deltaproteobacteria bacterium | Terrestrial, Soi (A0A1F9KFR3)
Betaproteobacteria bacterium | Terrestrial, Soil (A0A1F4F7S0)
TOBG NAT-575
MAG SAR202-VII (Ga0133547_10000004_0237)
MAG SAR202-VII (Ga0133547_10008610_0007)
TOBG SP-320
Ca. Rokubacteria bacterium 13_1_40CM_68_15 | Terrestrial, Soil (A0A1Q7D3F6)
Pseudonocardia thermophila | Terrestrial, Animal manure (A0A1M6UG12)
MAG SAR202-VII (Ga0133547_10000004_0238)
Ca. Entotheonella sp. TSY1 | Marine sponge symbiont (W4LAU4)
Caballeronia sordidicola | Terrestrial (UPI0004D02645)
Burkholderiaceae sp. | Freshwater, Drinking water (A0A1A9MY11)
Cupriavidus sp. UYMMa02A | Terrestrial, Mimosa magentea nodule (A0A1E4QSE4)
Burkholderiaceae bacterium 16 | Terrestrial, Soil (A0A0F0FCF7)
Pseudomonas azotifigens | Terrestrial, Compost material (UPI0003FE4389)
Sphingomonas mali | Estuary sediment (UPI0008299C14)
Skermanella stibiiresistens | Terrestrial, Soil (W9H036)
Sphingomonas paucimobilis GN_desZ_PE_4_SV_1 | Experimentally tested (Q7WYU8)
Streptomyces lushanensis | Terrestrial, Soil (UPI0008300D2F)
Streptomyces sp. AcH_505 | Terrestrial, Soil, Mycorrhizosphere (A0A0C2AZK0)
Pseudonocardia acaciae | Terrestrial, Acacia auriculiformis roots (UPI0004920F1A)
Rhodococcus yunnanensis | Terrestrial, Soil (UPI00093405DA)
Microbacterium azadirachtae | Terrestrial, Soil (A0A0F0LPY2)
Actinobacteria sp. M39 | Termite tissue (A0A178X6B3)
Sciscionella marina | Marine, sand sediments (UPI00037CB6FA)
0.2

Supplementary figure 4. Phylogenetic analysis of predicted 3-O-methylgallate dioxygenase pfam 02900) protein sequences identified in SAR202-VII-2. Method and description as described in Supplementary figure 2.

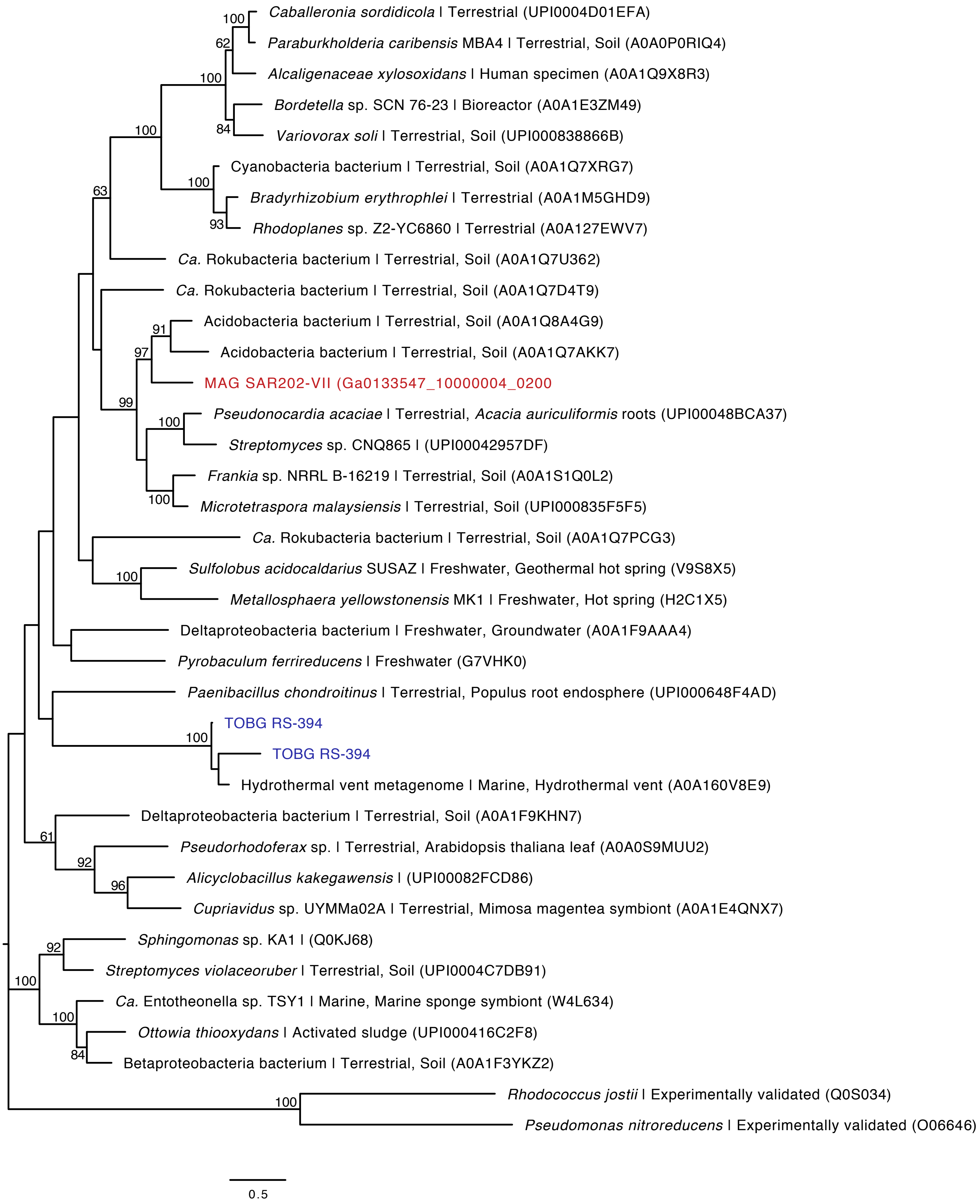

100
62
100
84
100
100
93
63
91
97
99
100
100
100
100
61
92
96
92
100
100
84
100
Caballeronia sordidicola | Terrestrial (UPI0004D01EFA)
Paraburkholderia caribensis MBA4 | Terrestrial, Soil (A0A0P0RIQ4)
Alcaligenaceae xylosoxidans | Human specimen (A0A1Q9X8R3)
Bordetella sp. SCN 76-23 | Bioreactor (A0A1E3ZM49)
Variovorax soli | Terrestrial, Soil (UPI000838866B)
Cyanobacteria bacterium | Terrestrial, Soil (A0A1Q7XRG7)
Bradyrhizobium erythrophlei | Terrestrial (A0A1M5GHD9)
Rhodoplanes sp. Z2-YC6860 | Terrestrial (A0A127EWV7)
Ca. Rokubacteria bacterium | Terrestrial, Soil (A0A1Q7U362)
Ca. Rokubacteria bacterium | Terrestrial, Soil (A0A1Q7D4T9)
Acidobacteria bacterium | Terrestrial, Soil (A0A1Q8A4G9)
Acidobacteria bacterium | Terrestrial, Soil (A0A1Q7AKK7)
MAG SAR202-VII (Ga0133547_10000004_0200
Pseudonocardia acaciae | Terrestrial, *Acacia auriculiformis* roots (UPI00048BCA37)
Streptomyces sp. CNQ865 | (UPI00042957DF)
Frankia sp. NRRL B-16219 | Terrestrial, Soil (A0A1S1Q0L2)
Microtetraspora malaysiensis | Terrestrial, Soil (UPI000835F5F5)
Ca. Rokubacteria bacterium | Terrestrial, Soil (A0A1Q7PCG3)
Sulfolobus acidocaldarius SUSAZ | Freshwater, Geothermal hot spring (V9S8X5)
Metallosphaera yellowstonensis MK1 | Freshwater, Hot spring (H2C1X5)
Deltaproteobacteria bacterium | Freshwater, Groundwater (A0A1F9AAA4)
Pyrobaculum ferrireducens | Freshwater (G7VHK0)
Paenibacillus chondroitinus | Terrestrial, Populus root endosphere (UPI000648F4AD)
TOBG RS-394
TOBG RS-394
Hydrothermal vent metagenome | Marine, Hydrothermal vent (A0A160V8E9)
Deltaproteobacteria bacterium | Terrestrial, Soil (A0A1F9KHN7)
Pseudorhodoferax sp. | Terrestrial, Arabidopsis thaliana leaf (A0A0S9MUU2)
Alicyclobacillus kakegawensis | (UPI00082FCD86)
Cupriavidus sp. UYMMa02A | Terrestrial, Mimosa magentea symbiont (A0A1E4QNX7)
Sphingomonas sp. KA1 | (Q0KJ68)
Streptomyces violaceoruber | Terrestrial, Soil (UPI0004C7DB91)
Ca. Entotheonella sp. TSY1 | Marine, Marine sponge symbiont (W4L634)
Ottowia thiooxydans | Activated sludge (UPI000416C2F8)
Betaproteobacteria bacterium | Terrestrial, Soil (A0A1F3YKZ2)
Rhodococcus jostii | Experimentally validated (Q0S034)
Pseudomonas nitroreducens | Experimentally validated (O06646)
0.5

Supplementary figure 5. Phylogenetic analysis of a predicted merged LigA/LigB fusion protein sequence identified in SAR202-VII-2. Method and description as described in Supplementary figure 2.

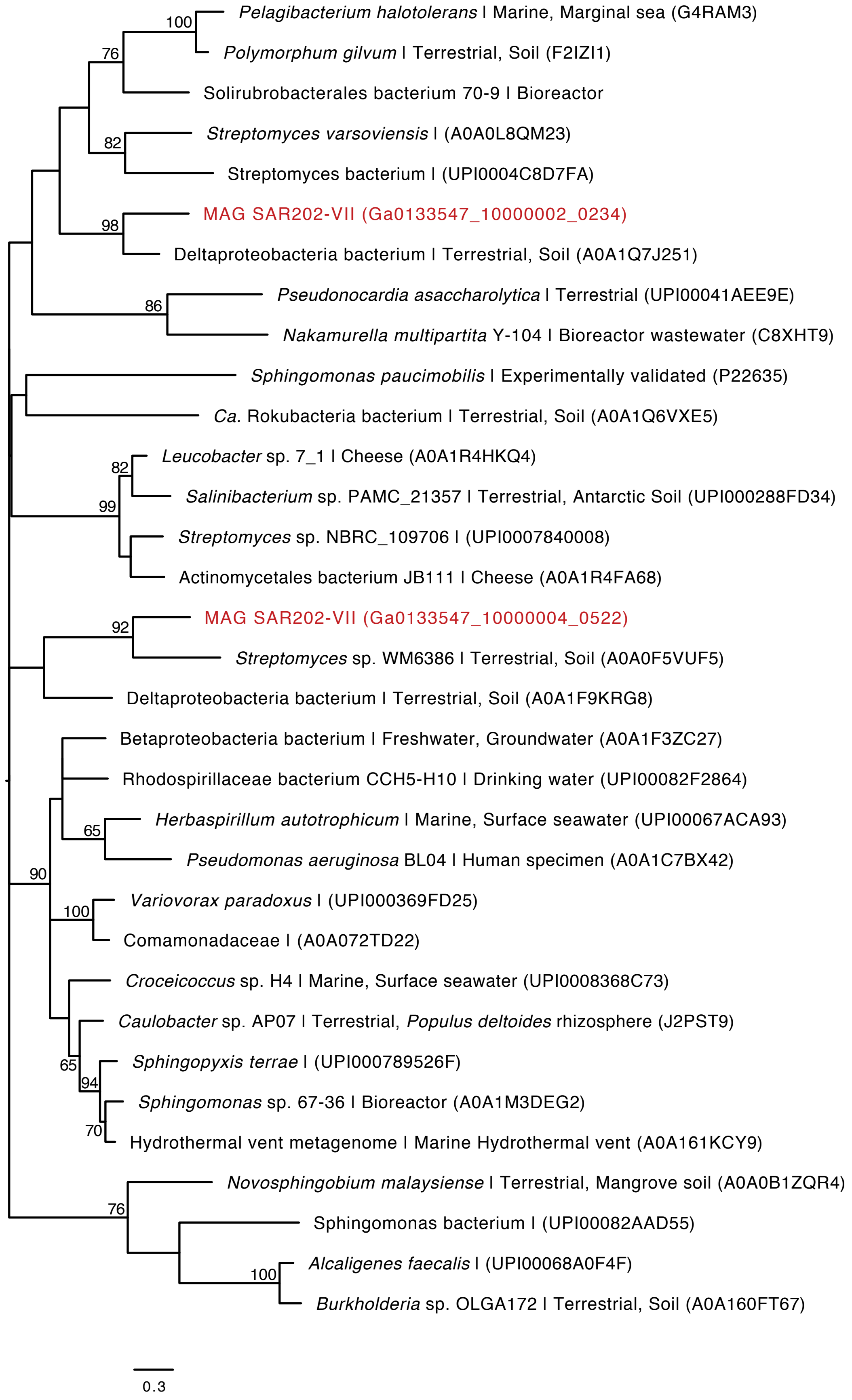

100
76
82
98
86
82
99
92
65
90
100
65
94
70
76
100
Pelagibacterium halotolerans | Marine, Marginal sea (G4RAM3)
Polymorphum gilvum | Terrestrial, Soil (F2IZI1)
Solirubrobacterales bacterium 70-9 | Bioreactor
Streptomyces varsoviensis | (A0A0L8QM23)
Streptomyces bacterium | (UPI0004C8D7FA)
MAG SAR202-VII (Ga0133547_10000002_0234)
Deltaproteobacteria bacterium | Terrestrial, Soil (A0A1Q7J251)
Pseudonocardia asaccharolytica | Terrestrial (UPI00041AEE9E)
Nakamurella multipartita Y-104 | Bioreactor wastewater (C8XHT9)
Sphingomonas paucimobilis | Experimentally validated (P22635)
Ca. Rokubacteria bacterium | Terrestrial, Soil (A0A1Q6VXE5)
Leucobacter sp. 7_1 | Cheese (A0A1R4HKQ4)
Salinibacterium sp. PAMC_21357 | Terrestrial, Antarctic Soil (UPI000288FD34)
Streptomyces sp. NBRC_109706 | (UPI0007840008)
Actinomycetales bacterium JB111 | Cheese (A0A1R4FA68)
MAG SAR202-VII (Ga0133547_10000004_0522)
Streptomyces sp. WM6386 | Terrestrial, Soil (A0A0F5VUF5)
Deltaproteobacteria bacterium | Terrestrial, Soil (A0A1F9KRG8)
Betaproteobacteria bacterium | Freshwater, Groundwater (A0A1F3ZC27)
Rhodospirillaceae bacterium CCH5-H10 | Drinking water (UPI00082F2864)
Herbaspirillum autotrophicum | Marine, Surface seawater (UPI00067ACA93)
Pseudomonas aeruginosa BL04 | Human specimen (A0A1C7BX42)
Variovorax paradoxus | (UPI000369FD25)
Comamonadaceae | (A0A072TD22)
Croceicoccus sp. H4 | Marine, Surface seawater (UPI0008368C73)
Caulobacter sp. AP07 | Terrestrial, Populus deltoides rhizosphere (J2PST9)
Sphingopyxis terrae | (UPI000789526F)
Sphingomonas sp. 67-36 | Bioreactor (A0A1M3DEG2)
Hydrothermal vent metagenome | Marine Hydrothermal vent (A0A161KCY9)
Novosphingobium malaysiense | Terrestrial, Mangrove soil (A0A0B1ZQR4)
Sphingomonas bacterium | (UPI00082AAD55)
Alcaligenes faecalis | (UPI00068A0F4F)
Burkholderia sp. OLGA172 | Terrestrial, Soil (A0A160FT67)
0.3

Supplementary figure 6. Phylogenetic analysis of predicted LigA dioxygenase (pfam 07746) protein sequences identified in SAR202-VII-2. Method and description as described in Supplementary figure 2.

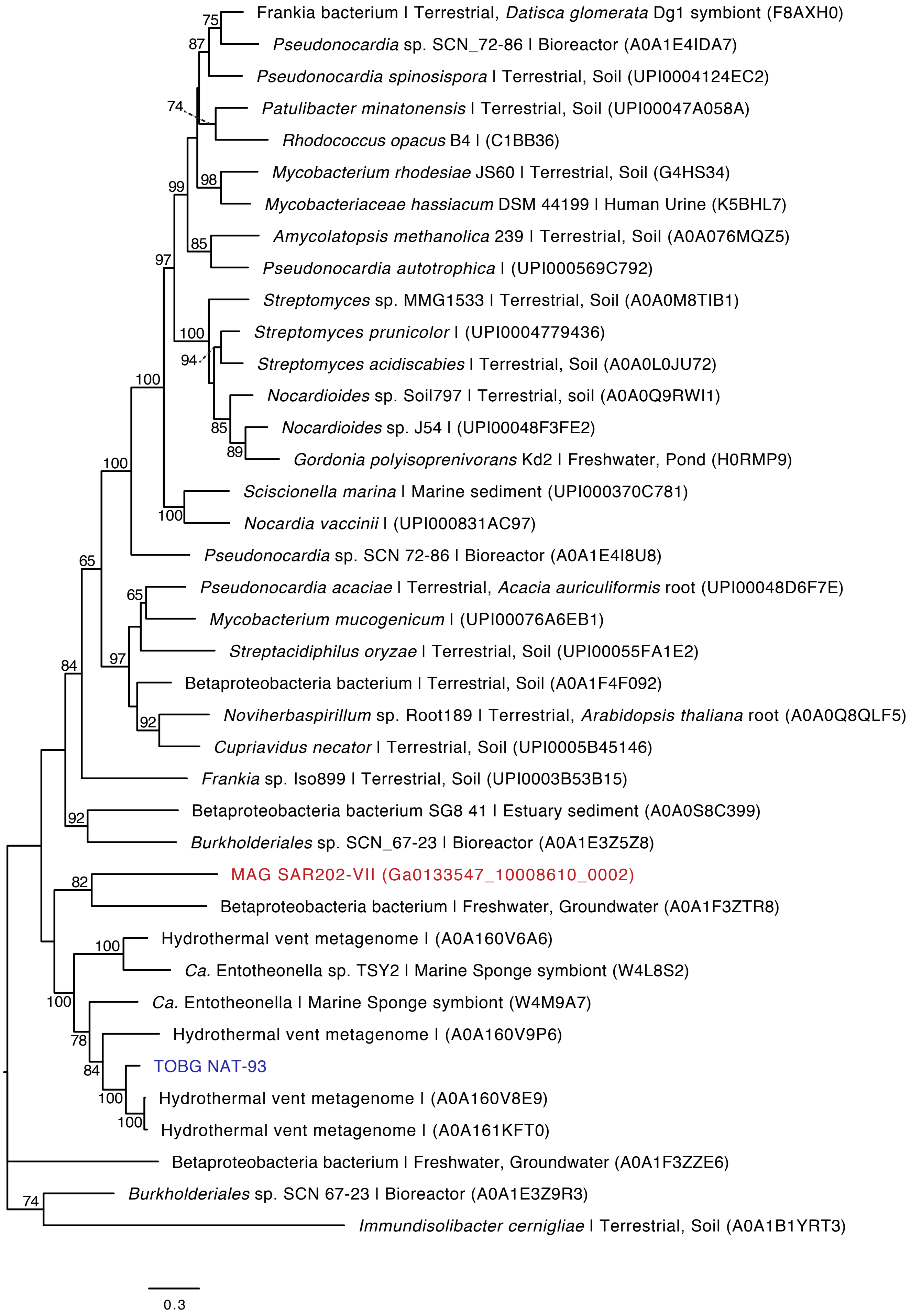

Frankia bacterium | Terrestrial, *Datisca glomerata* Dg1 symbiont (F8AXH0)
Pseudonocardia sp. SCN_72-86 | Bioreactor (A0A1E4IDA7)
Pseudonocardia spinosispora | Terrestrial, Soil (UPI0004124EC2)
Patulibacter minatonensis | Terrestrial, Soil (UPI00047A058A)
Rhodococcus opacus B4 | (C1BB36)
Mycobacterium rhodesiae JS60 | Terrestrial, Soil (G4HS34)
Mycobacteriaceae hassiacum DSM 44199 | Human Urine (K5BHL7)
Amycolatopsis methanolica 239 | Terrestrial, Soil (A0A076MQZ5)
Pseudonocardia autotrophica | (UPI000569C792)
Streptomyces sp. MMG1533 | Terrestrial, Soil (A0A0M8TIB1)
Streptomyces prunicolor | (UPI0004779436)
Streptomyces acidiscabies | Terrestrial, Soil (A0A0L0JU72)
Nocardioides sp. Soil797 | Terrestrial, soil (A0A0Q9RWI1)
Nocardioides sp. J54 | (UPI00048F3FE2)
Gordonia polyisoprenivorans Kd2 | Freshwater, Pond (H0RMP9)
Sciscionella marina | Marine sediment (UPI000370C781)
Nocardia vaccinii | (UPI000831AC97)
Pseudonocardia sp. SCN 72-86 | Bioreactor (A0A1E4I8U8)
Pseudonocardia acaciae | Terrestrial, *Acacia auriculiformis* root (UPI00048D6F7E)
Mycobacterium mucogenicum | (UPI00076A6EB1)
Streptacidiphilus oryzae | Terrestrial, Soil (UPI00055FA1E2)
Betaproteobacteria bacterium | Terrestrial, Soil (A0A1F4F092)
Noviherbaspirillum sp. Root189 | Terrestrial, *Arabidopsis thaliana* root (A0A0Q8QLF5)
Cupriavidus necator | Terrestrial, Soil (UPI0005B45146)
Frankia sp. Iso899 | Terrestrial, Soil (UPI0003B53B15)
Betaproteobacteria bacterium SG8 41 | Estuary sediment (A0A0S8C399)
Burkholderiales sp. SCN_67-23 | Bioreactor (A0A1E3Z5Z8)
MAG SAR202-VII (Ga0133547_10008610_0002)
Betaproteobacteria bacterium | Freshwater, Groundwater (A0A1F3ZTR8)
Hydrothermal vent metagenome | (A0A160V6A6)
Ca. Entotheonella sp. TSY2 | Marine Sponge symbiont (W4L8S2)
Ca. Entotheonella | Marine Sponge symbiont (W4M9A7)
Hydrothermal vent metagenome | (A0A160V9P6)
TOBG NAT-93
Hydrothermal vent metagenome | (A0A160V8E9)
Hydrothermal vent metagenome | (A0A161KFT0)
Betaproteobacteria bacterium | Freshwater, Groundwater (A0A1F3ZZE6)
Burkholderiales sp. SCN 67-23 | Bioreactor (A0A1E3Z9R3)
Immundisolibacter cernigliae | Terrestrial, Soil (A0A1B1YRT3)
75
87
74
98
99
85
97
100
94
100
85
89
100
100
65
65
97
92
84
92
82
100
100
78
84
100
100
74
0.3

Supplementary figure 7. Phylogenetic analysis of a predicted LigB dioxygenase (pfam 02900) protein sequence identified in SAR202-VII-2 (Ga0133547_100086102). Method and description as described in Supplementary figure 2.

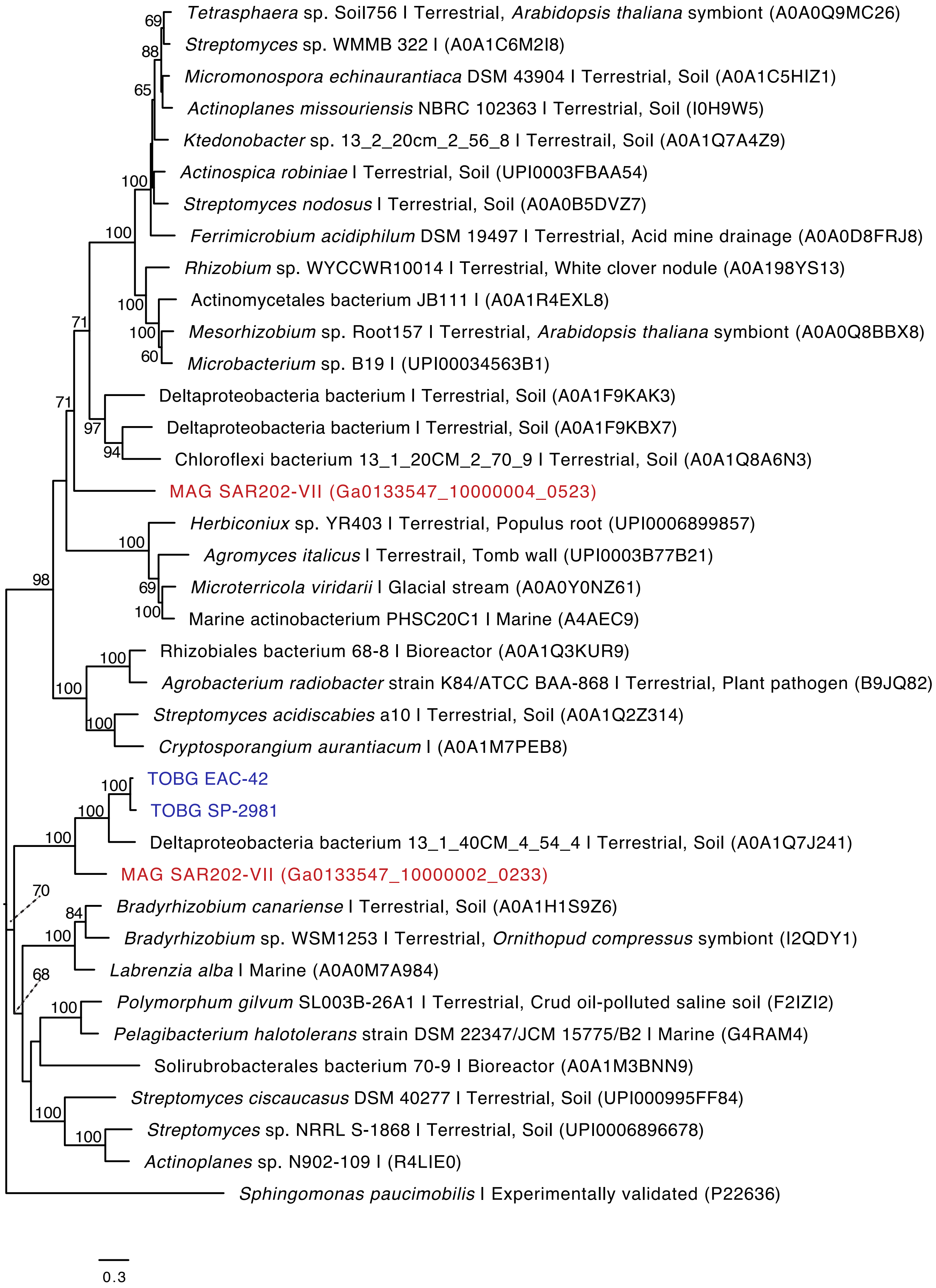
Tetrasphaera sp. Soil756 I Terrestrial, Arabidopsis thaliana symbiont (A0A0Q9MC26)
Streptomyces sp. WMMB 322 I (A0A1C6M2I8)
Micromonospora echinaurantiaca DSM 43904 I Terrestrial, Soil (A0A1C5HIZ1)
Actinoplanes missouriensis NBRC 102363 I Terrestrial, Soil (I0H9W5)
Ktedonobacter sp. 13_2_20cm_2_56_8 I Terrestrail, Soil (A0A1Q7A4Z9)
Actinospica robiniae I Terrestrial, Soil (UPI0003FBAA54)
Streptomyces nodosus I Terrestrial, Soil (A0A0B5DVZ7)
Ferrimicrobium acidiphilum DSM 19497 I Terrestrial, Acid mine drainage (A0A0D8FRJ8)
Rhizobium sp. WYCCWR10014 I Terrestrial, White clover nodule (A0A198YS13)
Actinomycetales bacterium JB111 I (A0A1R4EXL8)
Mesorhizobium sp. Root157 I Terrestrial, Arabidopsis thaliana symbiont (A0A0Q8BBX8)
Microbacterium sp. B19 I (UPI00034563B1)
Deltaproteobacteria bacterium I Terrestrial, Soil (A0A1F9KAK3)
Deltaproteobacteria bacterium I Terrestrial, Soil (A0A1F9KBX7)
Chloroflexi bacterium 13_1_20CM_2_70_9 I Terrestrial, Soil (A0A1Q8A6N3)
MAG SAR202-VII (Ga0133547_10000004_0523)
Herbiconiux sp. YR403 I Terrestrial, Populus root (UPI0006899857)
Agromyces italicus I Terrestrail, Tomb wall (UPI0003B77B21)
Microterricola viridarii I Glacial stream (A0A0Y0NZ61)
Marine actinobacterium PHSC20C1 I Marine (A4AEC9)
Rhizobiales bacterium 68-8 I Bioreactor (A0A1Q3KUR9)
Agrobacterium radiobacter strain K84/ATCC BAA-868 I Terrestrial, Plant pathogen (B9JQ82)
Streptomyces acidiscabies a10 I Terrestrial, Soil (A0A1Q2Z314)
Cryptosporangium aurantiacum I (A0A1M7PEB8)
TOBG EAC-42
TOBG SP-2981
Deltaproteobacteria bacterium 13_1_40CM_4_54_4 I Terrestrial, Soil (A0A1Q7J241)
MAG SAR202-VII (Ga0133547_10000002_0233)
Bradyrhizobium canariense I Terrestrial, Soil (A0A1H1S9Z6)
Bradyrhizobium sp. WSM1253 I Terrestrial, Ornithopud compressus symbiont (I2QDY1)
Labrenzia alba I Marine (A0A0M7A984)
Polymorphum gilvum SL003B-26A1 I Terrestrial, Crud oil-polluted saline soil (F2IZI2)
Pelagibacterium halotolerans strain DSM 22347/JCM 15775/B2 I Marine (G4RAM4)
Solirubrobacterales bacterium 70-9 I Bioreactor (A0A1M3BNN9)
Streptomyces ciscaucasus DSM 40277 I Terrestrial, Soil (UPI000995FF84)
Streptomyces sp. NRRL S-1868 I Terrestrial, Soil (UPI0006896678)
Actinoplanes sp. N902-109 I (R4LIE0)
Sphingomonas paucimobilis I Experimentally validated (P22636)
0.3

Supplementary figure 8. Phylogenetic analysis of predicted beta subunits of the protocatechuate 4,5-dioxygenase (pfam 02900) protein sequences identified in SAR202-VII-2 (Ga0133547_100086102). Method and description as described in Supplementary figure 2.
