# Supplementary material for: Genomic evidence for the degradation of terrestrial organic matter by pelagic Arctic Ocean Chloroflexi bacteria
Source: Commun Biol. 2018 Jul 5;1:90. doi: 10.1038/s42003-018-0086-7 (PMC6123686; doi:10.1038/s42003-018-0086-7)
Supplement: Supplementary file 2 — Description of additional Supplementary Infomation [file 42003_2018_86_MOESM2_ESM.docx]

Description of Additional Supplementary Items:

**Supplementary Data 1.** Twenty-five metagenomic data sets used for metagenomic fragment recruitment by best-hit reciprocal BLAST analysis.

**Supplementary Data 2.** Orthologous genes from aromatic compound degradation pathways identified in six Canada Basin Chloroflexi MAGs and 2 deep Ocean SAGs using Pathway Tools and ProteinOrtho, organized by enzymatic steps.

**Supplementary Data 3.** List of the 50 proteins used for the concatenated protein phylogeny in Supplementary figure 1.
